# Primary infected hydatid cyst of the thigh in a young lady; case report with literature review

**DOI:** 10.1016/j.amsu.2019.09.011

**Published:** 2019-09-27

**Authors:** Ahamd Mohammad Sharif Tahir, Alaa S. Bahjat, Ayad Ahmad Mohammed

**Affiliations:** a Cardiothoracic Surgeon, Duhok Directorate General of Health, Azadi Heart Center, Duhok City, Kurdistan region, Iraq; b Cardiothoracic Surgeon, Department of Surgery, College of Medicine University of Duhok, DUHOK, Kurdistan Region, Iraq; c General Surgeon, Department of Surgery, College of Medicine University of Duhok, DUHOK, Kurdistan Region, Iraq

**Keywords:** Hydatid disease, Anthelminthic medications, Musculoskeletal system, Infection, Intramuscular hydatid cysts

## Abstract

Hydatid disease present in certain parts of the world. Infection of the musculoskeletal system occur in less than 0.5%.

A 24-year-old lady had a painful mass in the inner aspect of the right thigh. MRI of the thigh showed a mixed signal intensity lesion measured about 65*100 mm, the mass was related to the muscle and the superficial femoral artery and its cavity had multiple septations.

During surgery an infected hydatid cyst of the muscle was found, evacuation was done with removal of the cyst. The patient was discharged next day and she received anthelminthic medications for 3 months.

Hydatid cyst of the muscles present with gradually enlarging mass or complications such as nerve compression, infection or rupture.

Treatment may be medical using anthelminthic medications. Complete surgical excision is the best surgical option; involvement of other organs should be excluded. Follow-up is recommended.

## Highlights

• Hydatid cysts could affect various body parts causing wide range of clinical presentations.
• Infection is one of the common complications of hydatid cysts.
• Surgery is the main treatment option in most of the cases.

## 1 Introduction

Hydatid disease is a human medical and veterinary problem in many parts of the world. The disease is mostly present in the developing countries, and transmitted by the fecal –oral route. The disease may be completely asymptomatic or may cause a variety of symptoms depending on the size and the presence or absence of complications. Infection in the musculoskeletal system is estimated to be less than 0.5%, this is because the cyst depends on high oxygen contents in the tissue for its growth and the muscle tissue has low oxygen contents and high lactic acid content which impedes the growth of the parasite [[1], [2], [3]].

The disease may be discovered accidentally or the patient may present with mass at the affected site. Hydatid cyst is classified into five main stages depending on the radiological appearance, the viability of the cyst, and the contents. Stage one and two indicate an active disease in which the contents of the cyst are clear and there may be undulating membrane (water-lily sign), stage three indicates that the cyst is entering a transitional stage and the integrity of the cyst is decreased by the host defense mechanism or the medications, and stage four and five indicates that the cyst is entering an inactive stage when the walls of the cyst become thick and calcified [4].

Serology may help in the diagnosis of hydatid disease but the final diagnosis is done by means of imaging. Ultrasound usually shows cyst lesion with single cavity or multiple cavities inside, ultrasound can also help in excluding hydatid cyst in other intra-abdominal organs, the best imaging information for muscle hydatid cysts are gained from the MRI which shows the location of the cyst, and its relations to the muscles and other structures, it also helps in excluding the presence of hydatid cyst in other parts of the body [1,2].

The work in this case report has been reported in line with the SCARE 2018 criteria [5].

## 2 Patient information

**Clinical findings:** A 24-year-old lady presented with gradually enlarging painful mass in the inner aspect of the upper thigh for the last 3 months, the patient noticed the mass accidentally during bathing. The mas was painless at the beginning but became painful with attacks of low grade fever mainly at the night. The patient had non-relevant past medical and past surgical histories.

During examination, the patient has normal general examination and examination of the mass showed a tender mass about 8*10 cm in the medial aspect of the right upper thigh with multiple enlarged ipsilateral inguinal lymph nodes.

The family history was negative for chronic illnesses. There was no history of chronic drug administration and the psychosocial history was negative.

**Fig. 1:**
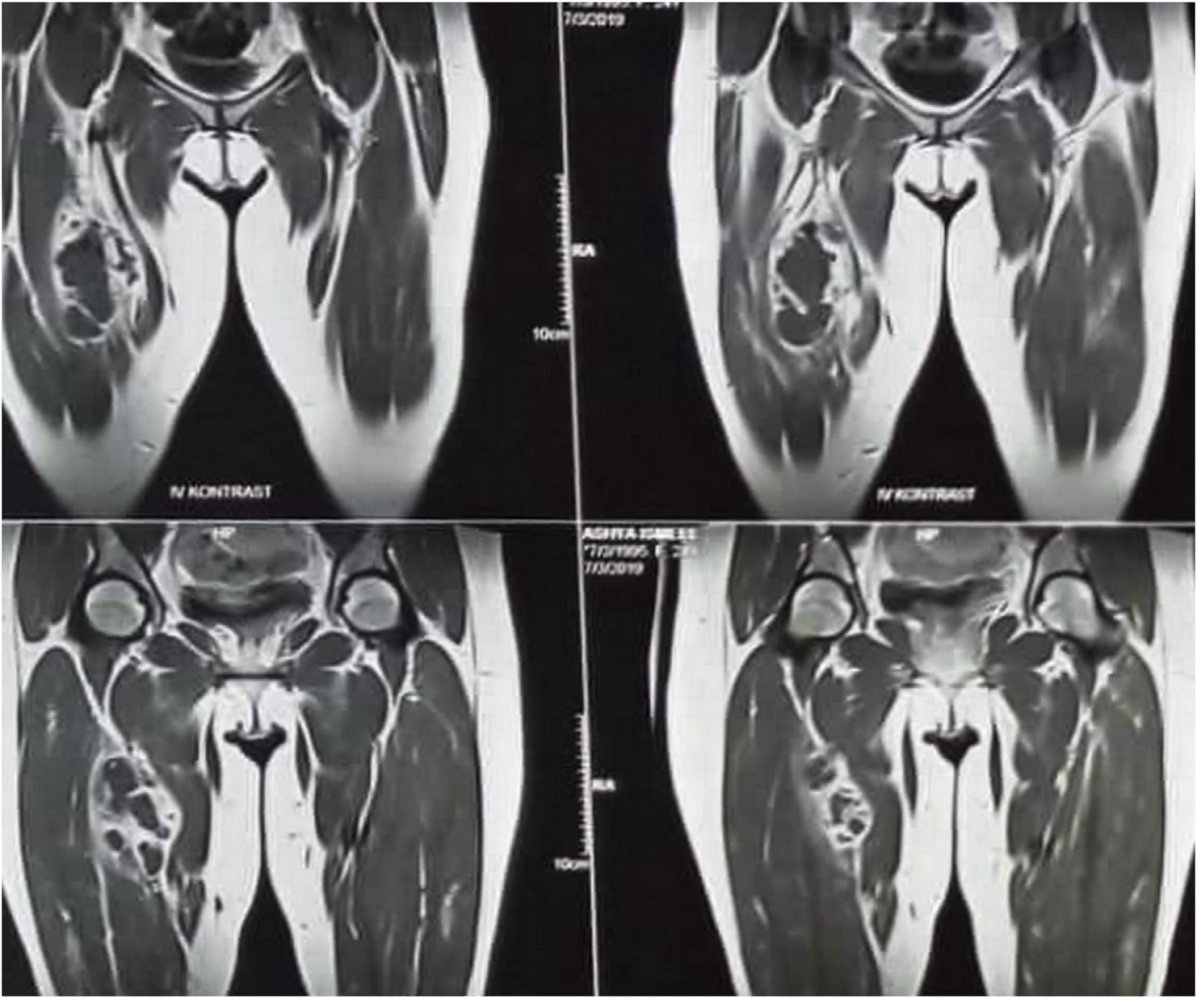
T1-weighted MRI study of the thigh showing a multi-loculated cystic lesion in the right thigh with multiple internal septations.

**Fig. 2:**
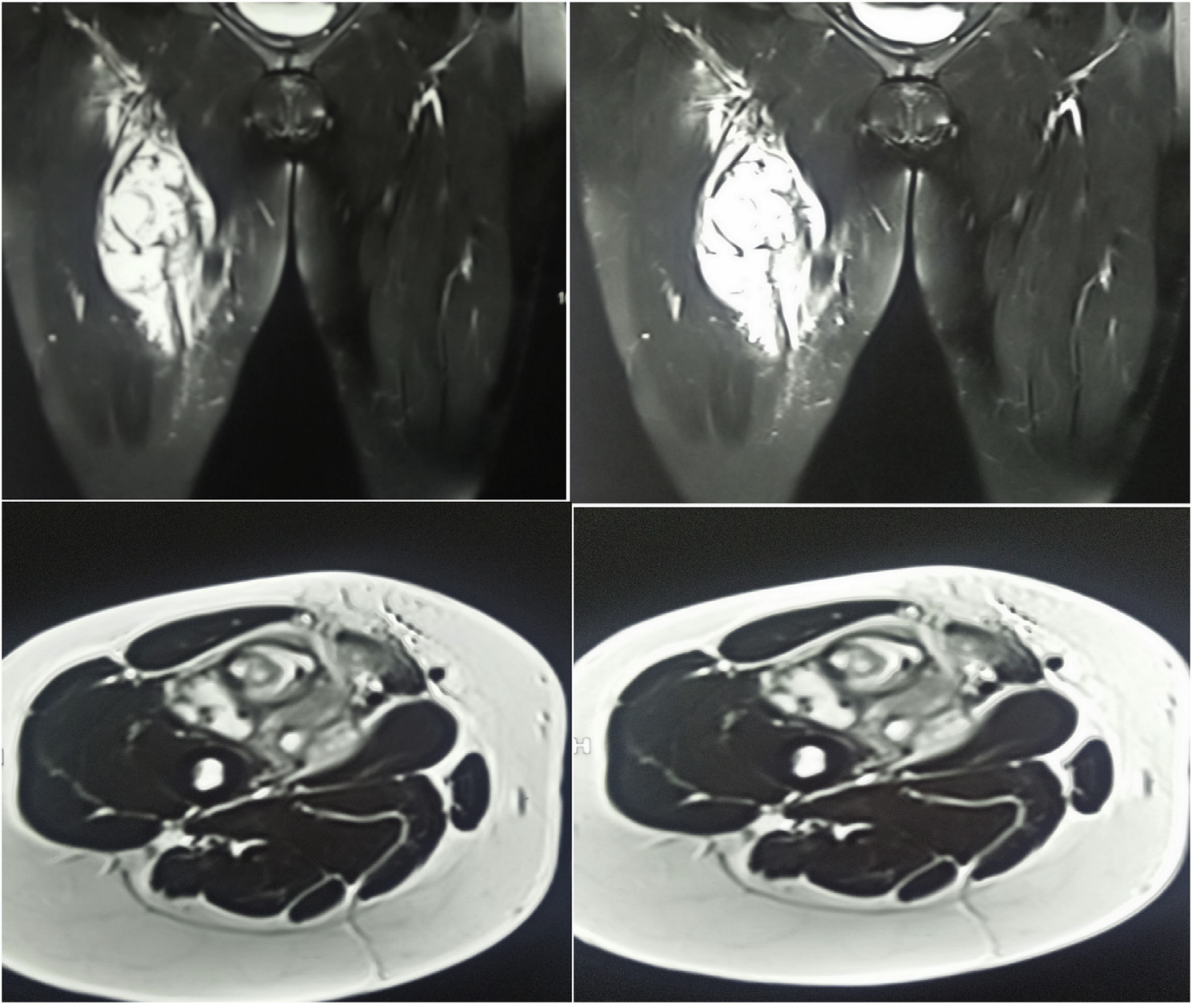
T2-weighted MRI study of the thigh showing a multi-loculated cystic lesion in the right thigh with multiple internal septations.

**Diagnostic assessment:** The white blood cells were elevated with raised inflammatory markers. MRI of the thigh showed a mixed signal intensity lesion located in the medial aspect of the right upper thigh and measured about 65*100 mm, the mass was related to the muscle compartment and appeared to be associated with the superficial femoral artery, there was no any associated bone abnormality and the lesion had multiple internal septations giving the possibility of either necrotic mass, cold abscess, or tumor. Fig. 1, Fig. 2.

**Fig. 3:**
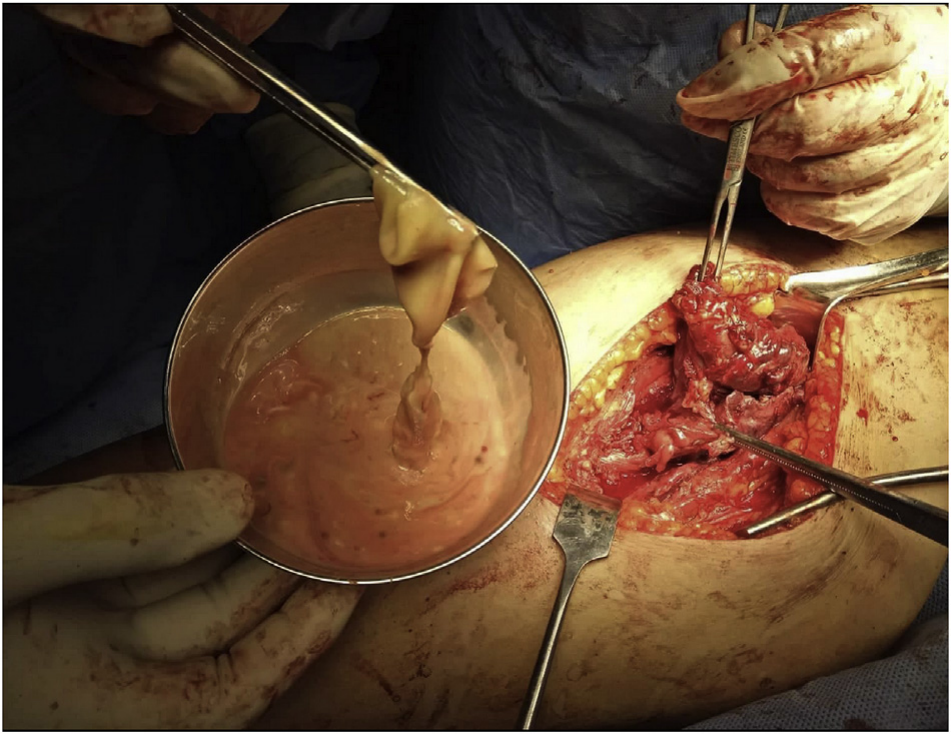
An intraoperative picture showing the hydatid cyst cavity contained pus and the hydatid cyst removed from the cavity.

**Therapeutic Intervention:** During exploration the lesion appeared to be an infected hydatid cyst of the muscle compartments of the medial aspect of the thigh, evacuation of the cavity was done with removal of the hydatid cyst, the cavity of the cyst was washed with chlorhexidine, low pressure suction drain was put in the cavity which was removed after 3 days. Fig. 3.

The operation was done by 3 specialist surgeons who were specialized in the field of the vascular and general surgery.

### 2.1 Follow-up and outcomes

The patient then was reevaluated to exclude hydatid cysts in other parts of the body by chest X-ray and abdominal ultrasound which showed no evidence of cysts in the chest and the abdomen.

The patient was discharged on the next day and she received anthelminthic medications for 3 months.

## 3 Discussion

Although hydatid cyst may occur mostly affects the liver and the lungs and infection in other organs may be secondary to infection of these sites but primary hydatid cyst of the muscles or some other rare anatomical places is well reported in literatures [1,6].

Many cases of muscle involvement are reported and the most common site of muscular involvement is the neck region followed by muscles of the trunk and then the root of the limbs, this is explained by the rich vascular supply in the above mentioned regions as hexacanth embryos are passed to various parts of the body through the blood stream [7].

Hydatid cyst of the muscles usually presents with a gradually enlarging mass which may be painful, some cysts may cause complications such as nerve compression causing pain, infection or rupture after trauma which may lead to allergic reaction [7].

Hydatid disease should be one of the differential diagnoses of any soft tissue mass in any part of the body particularly in patients from endemic areas [2].

Intramuscular hydatid cysts may cause diagnostic difficulties especially when the radiological appearance is atypical like in our case because the patient in this case presentation presented with infected cyst and the radiological appearance was confusing, so the patient was sent for tru cut biopsy to exclude soft tissue tumors [7].

The treatment of hydatid cyst depends on many factors such as the location of the cyst, the symptoms produced by the cyst, and the presence or absence of complications. The recommended treatment of hydatid cyst of the muscle is complete surgical resection of the entire cyst without opening the cavity to avoid spillage of the contents to decrease the recurrence rate. It is mandatary to exclude hydatid cyst in other parts of the body particularly the lungs when the surgery is done under general anesthesia [8,9].

Medical therapy with anthelminthic medications may be used with variable responses, calcified cysts usually doesn't respond to medical treatment, medical therapy may be also used preoperatively and postoperatively to decreases the recurrence rate [3].

## Patient perspective

I thought that I had a tumor in my thigh and I was worried too much about that, after surgeries my worries had gone but I am worried now about the recurrence of the development of cysts elsewhere in my body.

## Informed consent

An informed written consent was taken from the patient for reporting this case and the accompanying images.

## Funding source

The authors are the source of the funding.

## Provenance and peer review

Not commissioned, externally peer reviewed.

## Ethical approval

No ethical committee approval was needed; consent have been taken from the family to report their findings.

## Sources of funding

No source of funding other than the authors.

## Author contribution

The surgeon who performed the procedure: Dr Ahamd Mohammad Sharif Tahir, Dr Alaa S. Bahjat and Dr Ayad Ahmad Mohammed.

Study design, writing, and the final approval of the manuscript: Dr Ayad Ahmad Mohammed.

## Trial registry number

N/A.

## Guarantor

Dr Ayad Ahmad Mohammed.

## Declaration of competing interest

No conflicts of interest present.
